# Minimally invasive thermal cauterotherapy (Agnikarma) in grade V rectal prolapse: a unique case report

**DOI:** 10.3389/fmed.2026.1761909

**Published:** 2026-04-10

**Authors:** Sruthi Chenganakattil, Soumya Mohan, Aparna Thilak, Shyam Vinayan

**Affiliations:** Department of Shalya Tantra, Amrita School of Ayurveda, Kollam, Kerala, India

**Keywords:** Agnikarma, case report, Gudabhramśa, procidentia, rectal prolapse

## Abstract

**Introduction:**

Rectal prolapse occurs when layers of the rectum protrude through the anus, which is classified as partial or complete based on the extent of the tissue involvement. The condition has a yearly rate of 2.5% per 100,000 individuals, with a higher incidence in those over 50 due to muscle weakness. Surgical intervention is often necessary to repair the prolapse, manage incontinence, and address underlying factors.

**Methodology:**

In this case report, a para-surgical method known as Agni Karma was used, which applies localized heat to promote healing and reduce tissue prolapse. The condition was assessed using the Oxford Radiological Rectal Prolapse Rating System.

**Result and conclusion:**

The patient was initially rated as grade five, indicating an external prolapse. Following the Agni Karma treatment, the follow-up showed significant improvement, reducing the grade to three.

## 1 Introduction

Rectal prolapse refers to a condition in which the rectum slides out through the anal canal, essentially turning inside out. When the prolapse is external and involves the full thickness of the rectal wall, it typically appears as multiple circular folds projecting outside the anus. This form is different from haemorrhoidal prolapse, which is identified by its distinct radial folds (1). Although the exact reason for rectal prolapse is still not clearly understood (2). It is known to occur more often in women. Several predisposing factors have been identified, including weakness or damage to the pelvic floor, problems with connective tissue or the anal sphincter, chronic constipation, increased intra-abdominal pressure, pregnancy, obesity, and trauma to the perineal region (2). Epidemiologically, rectal prolapse is seen in about 2.5 cases per 100,000 people each year, with a higher frequency reported after the fifth decade of life (3). Diagnosis is usually made through a detailed medical history and a thorough anorectal examination. Depending on the clinical presentation, further investigations may also be required. Additional tests, including colonoscopy and barium enema, will be performed to confirm the diagnosis and identify any other relevant pathologies (4). Various surgical interventions like rectopexy, Delorme's operation, perineal proctosigmoidectomy are in practice depending upon the condition of the patient (5). The decision regarding surgery is influenced by several factors, including the patient's age, gender, level of incontinence, surgical risks, and skill level.

As per *Sushrutacharya, Gudabhramśa* is the term used for rectal prolapse, which is explained under *Kṣudraroga*, while explaining the diagnosis and treatment of minor disease (6). In Charaka Samhita and Aṣṭānga Hridaya, this condition is depicted as a troubling complication resulting from *Atisāra*, or chronic diarrhea, and is often seen as a dire symptom of excessive purgation (*Atiyog*a of *virechana*). Prolonged diarrhea and frequent bowel movements can weaken the rectum, leading to rectal prolapse, which causes discomfort. This case report outlines a treatment plan that involves internal medications, anal infiltration, and para-surgical management with Agni karma. This approach aims to restore the integrity of the rectum and to prevent the disease from recurring.

## 2 Materials and methods

### 2.1 Patient information

A 63-year-old female patient came to the outpatient department (OPD) with a 2-year history of experiencing a mass that protrudes from the rectum during defecation. She had to manually reduce the mass once it came out and was also facing difficulties with passing stools and experiencing itching around the anus. She had been diagnosed with partial rectal prolapse and was posted for surgical intervention at a modern hospital. However, during the administration of a pre-operative enema, the patient sustained iatrogenic burns, which resulted in the postponement of her surgical procedure. The patient had a prior history of vaginal hysterectomy. There was no significant family history noted. Before the administration of *Agnikarma*, the patient underwent 6 months of conservative management, which enhanced the effectiveness of *Agnikarma*.

### 2.2 Examination

#### 2.2.1 Ano rectal examination

Inspection:

- Circular folds of mucosal tissue with bright red color are seen.
- On coughing: Circumferential prolapse was more evident, which was lined with mucous membrane discharging mucus.
- In squatting position: The patient was asked to sit in a squatting position so as to increase intra-abdominal pressure, thereby relaxing the entire mass of the rectum, which facilitated the thorough examination of the prolapsed mass.
- Prolapsed mass was seen outside the anal verge with a length of 7 cm. Palpation: non-tender soft mass.

#### 2.2.2 Per rectal examination

Loose elastic superficial mucosa was palpated superficially along with consecutive circumferential fibrotic folds.

#### 2.2.3 General examination

Systemic examination, General Examination, *Dashavidha pareeksha*, and *Ashtasthana pareeksha* presented in Table 1.

**Table 1:** *Ashtasthana pareeksha*, systemic and general examination.

Systemic examination	General examination	*Dasavidha pareeksha*	*Ashtasthana pareeksha*
Cardiovascular system: not affected Respiratory system: not affected Alimentary system: affected Genitourinary system: not affected Locomotor system: not affected Hemopoietic and reticulo-endothelial system: not affected	Patient is oriented, co- operative, afebrile Blood pressure:130/90 mmHg Respiratory Rate:18/min Pulse rate:72 beats/min	*Dooshyam: Mamsa, Raktha Desam: Anupa Balam: Madhyama Kalam: Varsha Analam: Madhyamam Prakruthi: Vathapitha Vayah: Vrudha Satwam: Madhaymam Sathmyam: Sarvarasa Ahara: Madhyamam*	*Nadi: Dhrutha Moothram: Prakrutha Malam: Vaikrutha Jihva: Anupaliptha Sabdha: Spashta Sparsha: Anushnaseetha Druk: Vyaktham Akriti: Madhyamam*

### 2.3 Procedure of Agnikarma

➣ Pre-operative (See Table 2).

- Detailed procedure was explained to the patient.
- Written consent was taken.
- The patient was made to lie down in a lithotomy position.
- The anal canal and perianal region were painted with an aseptic solution and draping was done.

**Table 2:** Therapeutic intervention.

Date	Treatments given
12/09/2023 to 18/09/2023	Anal infiltration of *Pippalyādi anuvasana tailam* 50 ml—Once daily
29/04/2024 to 04/05/2024	*Chiruvilwadi kashayam* 50 ml twice daily before food *Kankayana vati* 1 tablet twice daily before food Tab. Pilex 1 tablet twice daily before food
06/06/2024	*Agnikarma* with cautery
06/04/2025	Follow-up visit

➣ Operative procedure

- A field block was given by infiltrating 7.5 ml of 2% lignocaine with adrenaline over the perianal region.
- Lord's dilation was performed to widen the surgical area for Agnikarma.
- Temperature: monopolar diathermy probe was set to coagulation mode at 30 W.
- Duration: for 3 s at each application point.
- Number of applications: single sitting.
- The prolapsed rectum was identified and pulled tightly downwards with Babcock forceps, allowing the redundant rectal wall to be incorporated into the prolapsed segment.
- Superficial to deep cauterization was performed from the mucocutaneous (anal verge) junction up to the dentate line, approximately 3 cm from the anal verge. The cauterization is applied in a circumferential manner, maintaining a 1 cm gap between each cauterized band, with a total of three circumferential cauterization segments.
- A single superficial circumferential cauterization was performed at the level of the palpable anorectal ring under per-rectal guidance using the left index finger, with careful attention to avoid injury to the puborectalis muscle.

➣ Post-operative

- Haemostasis was attained.
- The area was packed with *Sathadhoutha Ghritham*.

### 2.4 Timeline of events

**Figure 1:**
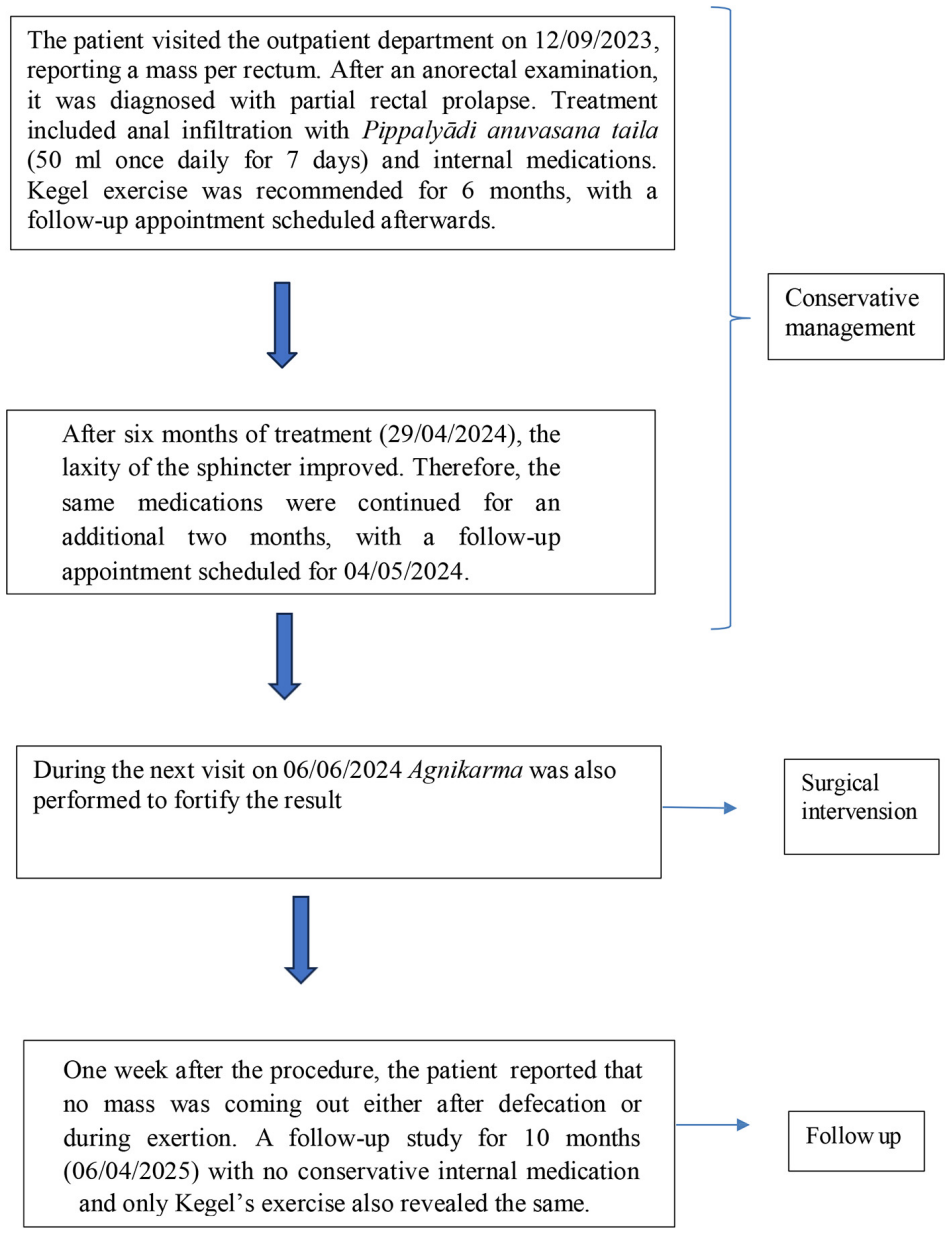
Timeline of events.

Figure 1 illustrates the timeline of events.

### 2.5 Follow up and outcome

➣ Grade of rectal prolapse.
  Before and after comparison of grade of rectal prolapse using Oxford
Radiological Rectal Prolapse Rating System^a^ (see Table 3).
➣ Wexner constipation score (see Table 4).
➣ VAS itch scale (see Table 5).

**Table 3:** Prolapse rating system.

Date of observation	Grade
12/09/2023	Grade 5
29/04/2024	Grade 4
04/05/2024	Grade 4
06/06/2024	Grade 3
06/04/2025	Grade 3

^a^Supplementary material.

**Table 4:** Before and after comparison of constipation score.

Date of observation	Score
12/09/2023	26
29/04/2024	20
04/05/2024	15
06/06/2024	15
06/04/2025	3

^b^Supplementary material.

**Table 5:** Before and after comparison of Pruritis ani.

Date of observation	Score
12/09/2023	7
29/04/2024	5
04/05/2024	5
06/06/2024	5
06/04/2025	1

^c^Supplementary material.

## 3 Result

**Figure 2:**
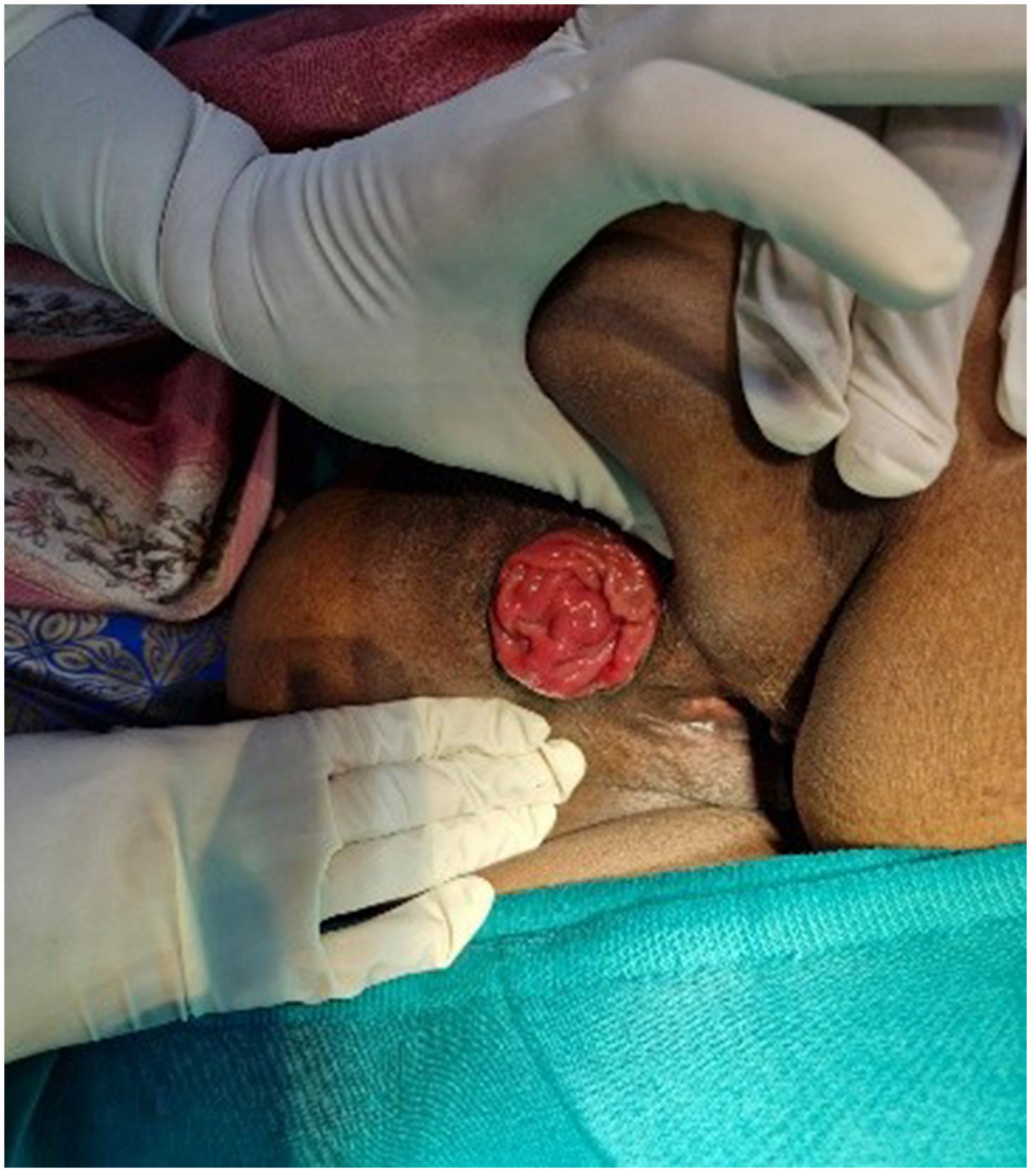
Photograph showing the circumferential prolapse of rectal mucosal folds during anorectal examination in the left lateral position (as on 12-09-2023).

**Figure 3:**
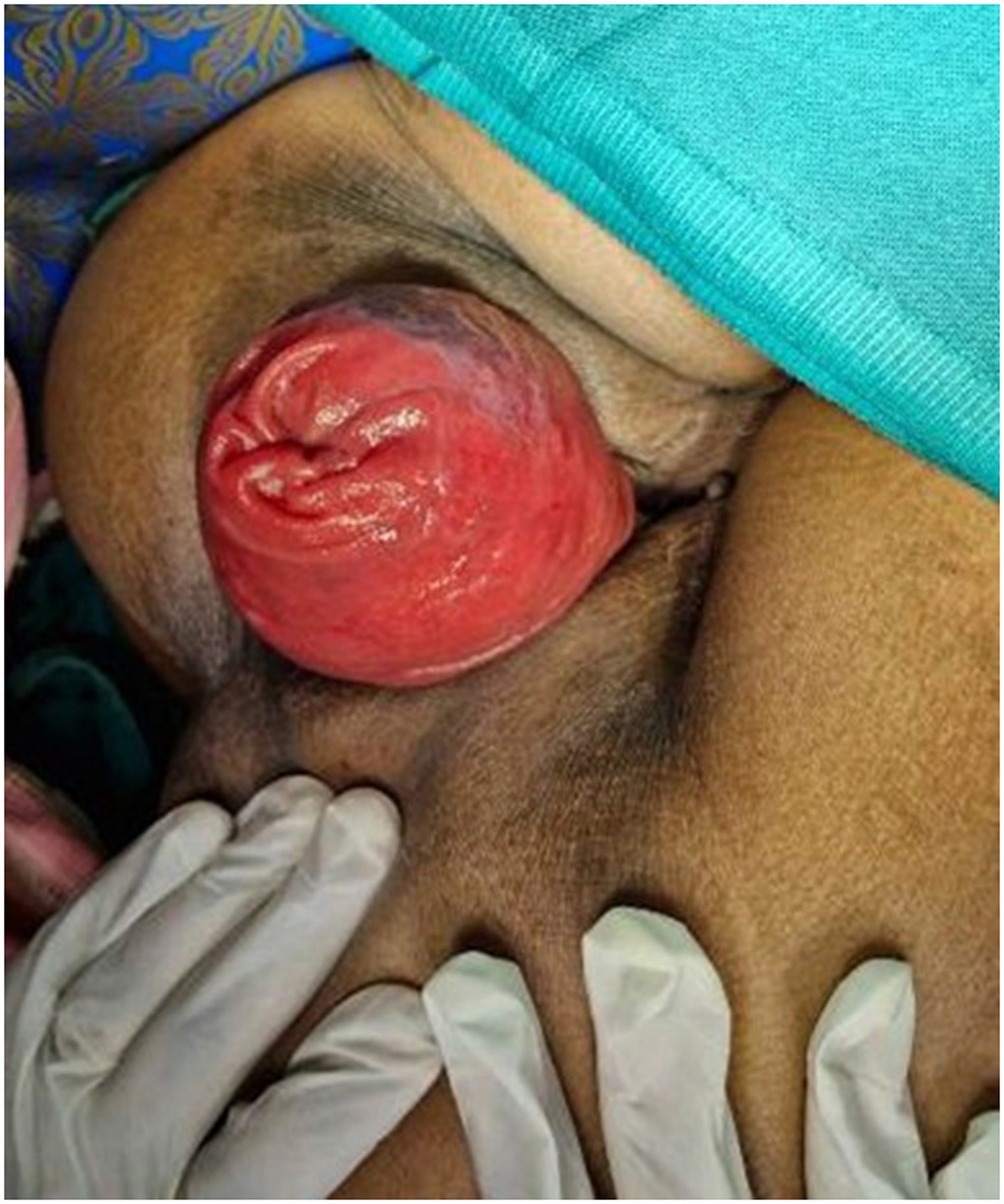
A photograph showing a prolapsed rectal mass protruding through the anal verge during anorectal examination on straining (as on 12-09-2023).

**Figure 4:**
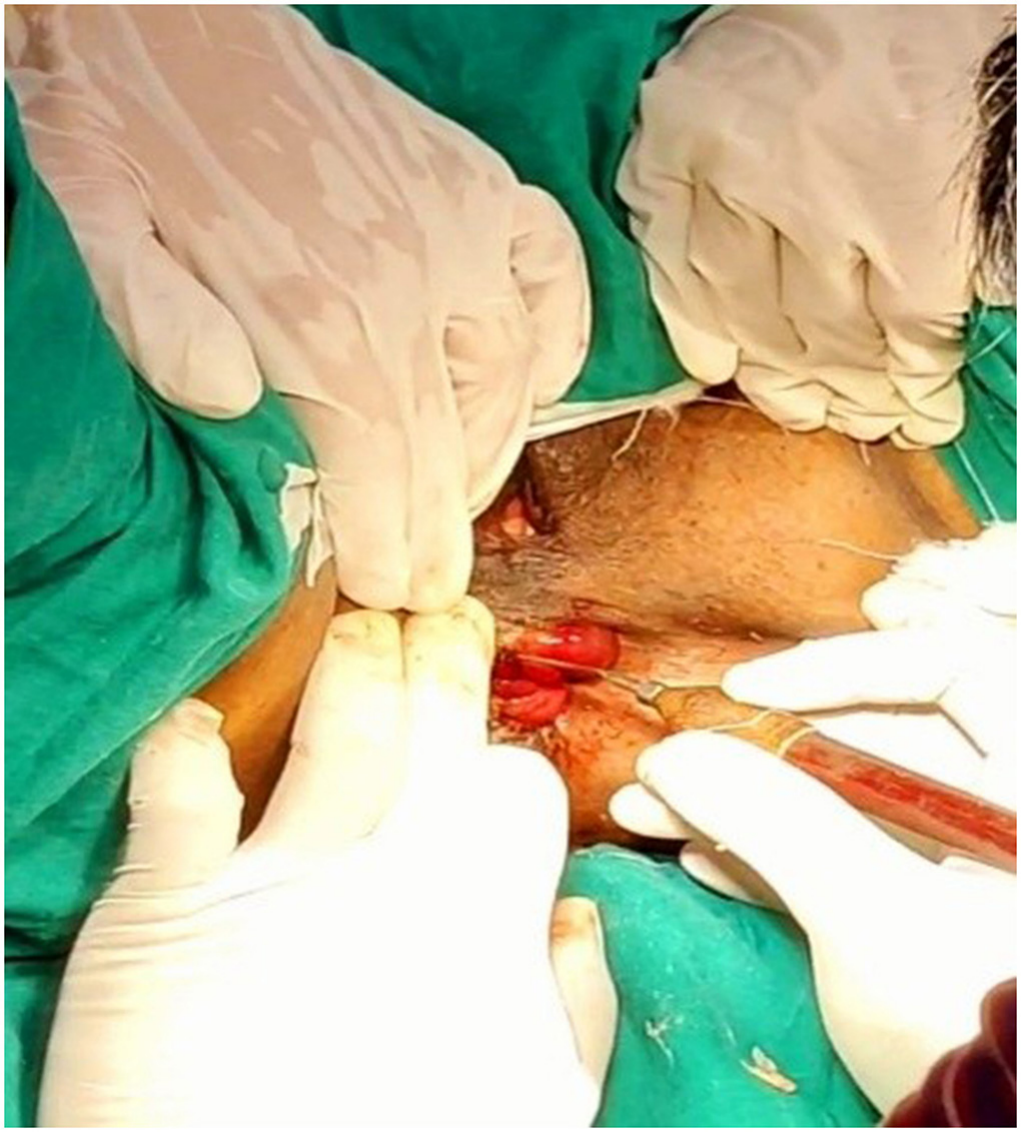
Photo taken during the procedure of Agnikarma for rectal prolapse done on 06-06-2024.

**Figure 5:**
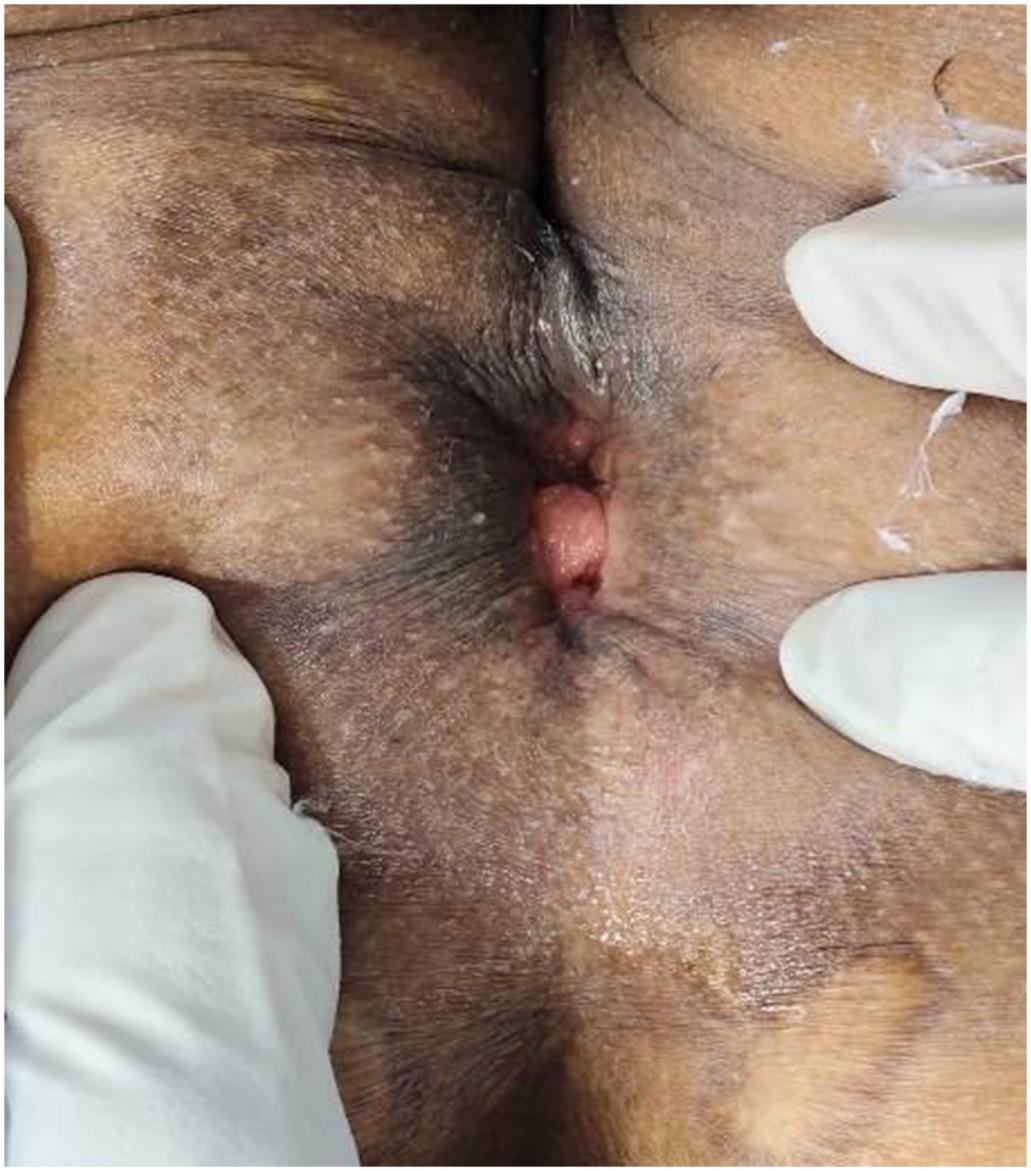
Follow-up clinical photograph (06-04-2025) taken during anorectal examination showing rectal prolapse at the anal verge.

The patient experienced symptomatic relief following treatment. The severity of the rectal prolapse improved from grade 5 to 3. The Wexner constipation score decreased from 26 to 15. Additionally, significant relief from itching was observed, with a reduction in severity from level 7 to 1 (Figures 2–5).

### 3.1 Statistical analysis

Data was analyzed using the statistical package SPSS 26.0 (SPSS Inc., Chicago, IL, USA) and level of significance was set at *p* < 0.05. Descriptive statistics was performed to assess the mean and standard deviation of the respective groups.

Table 6 presents the within-group comparison of outcomes during the conservative management phase (T1–T4). There was a progressive improvement in all measured variables over time, with the grade of rectal prolapse reducing from 5 to 4 (change −1), Wexner constipation score decreasing from 26 to 15 (change −11), and VAS itch score improving from 7 to 5 (change −2). These findings indicate clinically meaningful improvement with conservative therapy, particularly in constipation severity.

**Table 6:** Conservative management.

Variable	T1	T4	Change (T4–T1)	Interpretation
Grade of rectal prolapse	5	4	−1	Mild improvement
Wexner constipation score	26	15	−11	Marked improvement
VAS itch score	7	5	−2	Moderate symptom relief

Table 7 presents the within-group comparison during the surgical management phase (T1–T2). Following surgery, there was a marked improvement across all recorded outcome measures.

**Table 7:** Surgical management.

Variable	T1	T2	Change (T2–T1)	Interpretation
Grade of rectal prolapse	4	3	−1	Further improvement post-surgery
Wexner constipation score	15	3	−12	Very large reduction in constipation severity
VAS itch score	5	1	−4	Substantial reduction in itching

The grade of rectal prolapse showed a further reduction from 4 to 3 (change −1), indicating enhanced anatomic correction post-operatively. There was a substantial reduction in constipation severity, with the Wexner score improving dramatically from 15 to 3 (change −12), representing the greatest functional gain observed across all measured parameters.

Similarly, subjective symptom burden showed significant relief, reflected by the decrease in VAS itching score from 5 to 1 (change −4).

Conservative management (T1–T4) produced gradual improvement in prolapse grade, constipation severity, and itching, while surgical intervention (T1–T2) led to a more marked and rapid reduction in all outcome measures (Table 8).

**Table 8:** Effect direction summary.

Measure	Conservative change	Surgical change	Difference
Grade	−1	−1	Equal reduction
Wexner	−11	−12	Greater improvement post-surgery
VAS	−2	−4	Greater improvement post-surgery

**Figure 6:**
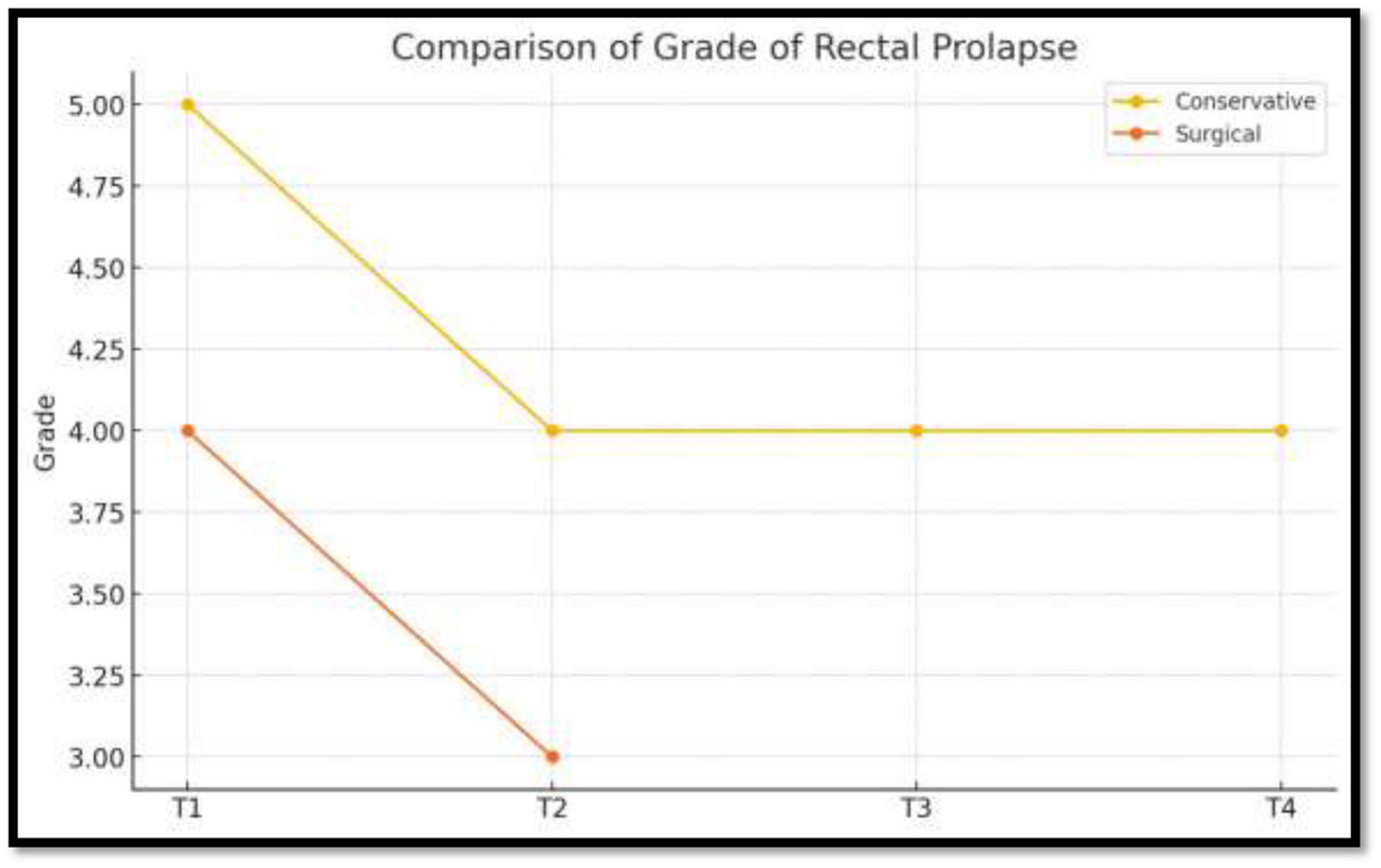
Comparison of grade of rectal prolapse.

**Figure 7:**
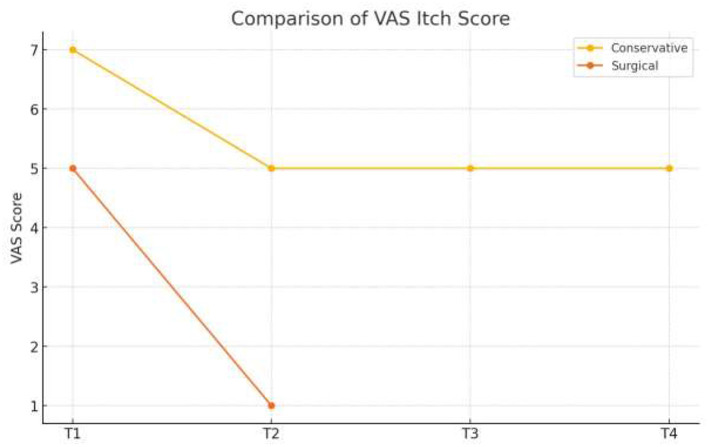
Comparison of VAS itch scale.

**Figure 8:**
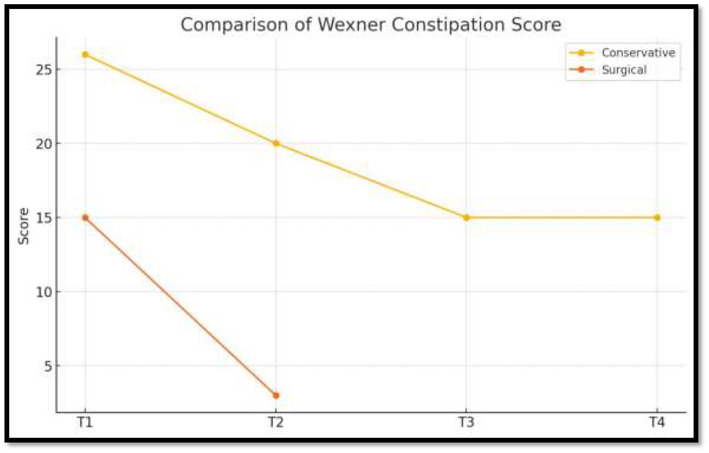
Comparison of Wexner constipation score.

The direction of change favored surgical management, which demonstrated greater improvement clinically across all variables. Over 9 months of conservative treatment, a grade 5 prolapse was reduced to grade 4. After the *Agnikarma* procedure, it dropped further to grade 3 almost immediately. While the statistical differences between conservative and surgical treatments are minor and technical, the clinical significance is clear when looking at the time taken to achieve these results. Furthermore, during follow-up without any internal medication, the prolapse maintained its grade 3 status, demonstrating the effectiveness of the *Agnikarma* procedure (Figures 6–8).

## 4 Discussion

### 4.1 Need of the study

Although surgery is the mainstay of treatment for rectal prolapse, a small proportion of patients continue to experience persistent or recurrent symptoms despite successful operations and proper bowel regulation. Perineal procedures such as Delorme's procedure and Altemeier procedure are commonly performed, particularly in elderly or high-risk patients, as they can be done under local or regional anesthesia and are associated with shorter hospital stay (7).

However, perineal approaches are reported to have higher recurrence rates and an increased risk of fecal incontinence. Delorme procedures show particularly high recurrence rates, and concerns remain regarding long-term durability and functional outcomes (8). The Altemeier procedure, which involves full-thickness excision of the rectum and sigmoid colon, carries the additional risk of serious complications such as anastomotic leak, often related to inadequate perfusion, oedema, or raised intraluminal pressure (9).

Given these limitations and the ongoing controversy regarding the most appropriate surgical option, there is a need for safer, minimally invasive alternatives. This case report highlights a minimally invasive procedure that may be suitable even for geriatric patients or those unfit for general or spinal anesthesia.

### 4.2 Mode of action of *Pippalyādi anuvasana tailam*

*Pippalyādi Anuvasana Taila* is described in the *Aṣṭāṅga Hṛdaya Cikitsāsthāna*, 8th chapter *(Arśaḥ Cikitsā Adhyāya)*, where its mode of administration is specified as Anuvasana Vasti. Anal infiltration of the medicated oil using an infant feeding tube may be considered a modified form of *Anuvasana Vasti*, as it potentially minimizes trauma associated with the conventional enema nozzle. Anal infiltration with *Pippalyādi anuvasana tailam* provides lubrication (*Snehana*) and softening of the tissues and stool in a way that makes them more pliable and reduces friction, which eases the passage of stools and reduces the strain that can worsen prolapse (10). This may reduce discomfort and prevent further prolapse or damage to the prolapsed tissues by decreasing pressure on the anal sphincter and surrounding areas. Oil infiltration may nourish the mucosal and muscular tissues of the anal and rectal areas. This nourishment helps improve the structural integrity of the rectal and anal muscles, potentially improving the support of the rectum. Improving the tone of the anal sphincter can play an important role in preventing further prolapse. When the sphincter becomes stronger and better supported, the rectum is less likely to slip downwards, which in turn helps with continence and may lower the chances of recurrence.

### 4.3 Mode of action of *Agnikarma*

*Agnikarma* is a para-surgical technique described in traditional Ayurvedic practice. The procedure is based on the controlled application of heat to selected points on the body. It is usually done as an outpatient procedure under local anesthesia and is generally considered safe, simple, and affordable.

The heat applied during *Agnikarma* leads to a focused contraction of tissues, later followed by mild fibrosis. This combination can help reduce excess or lax tissue, which may slow down the progression of prolapse. By shrinking and tightening the treated area, the mechanical strain on the rectum decreases, offering indirect support in managing the condition.

Another important effect is the stimulation of healing in the surrounding tissues of the anal canal and rectum. As these tissues gradually repair and regain strength, their tone improves, which can reduce the chances of repeated descent or future prolapse.

### 4.4 Role of exercises in the prevention of rectal prolapse

Preventing rectal prolapse largely depends on maintaining regular bowel habits, and this often starts with simple adjustments in day-to-day diet and lifestyle. Strengthening the anal sphincter is another important aspect of prevention. Pelvic-floor exercises, though easy to overlook, gradually improve the tone and stability of the muscles in the pelvic region. With regular practice, these exercises tend to enhance bowel control and support the rectum more effectively, which may help reduce the likelihood of prolapse recurrence (11).

## 5 Conclusion

- This method of management may be considered as a viable alternative to conventional surgical approaches. Because it relies on simple, minimally invasive measures and the patient experienced minimal post-procedural pain, short hospital stay, with favorable results. Further studies with a larger sample size are required to confirm the result.
- Exercises that focus on the pelvic floor—such as Kegel's exercise- have a helpful role in improving anal sphincter function and supporting overall rectal health.

## 6 Patient perspective

“I had been struggling for quite some time with a mass that would slip out of my anal canal after passing stools. Each time it happened, I had to gently push it back, which was both uncomfortable and embarrassing. Along with this, I often felt itchy and never quite satisfied after evacuation, as if something was still left inside. Walking or even coughing made the discomfort worse.

Eventually, I was told that it was rectal prolapse. Surgery was suggested, but I was unable to go ahead with it because I had suffered burns following an enema. During this period, I decided to try Ayurvedic management. Over the course of the treatment, I noticed a clear improvement—the mass stopped coming out, the discomfort reduced, and I began to feel more at ease in my daily activities. Overall, I am genuinely relieved and grateful to the medical team for the care I received.”

## Data Availability

The original contributions presented in the study are included in the article/Supplementary material, further inquiries can be directed to the corresponding author.
