# Supplementary figures and images for: Minimally invasive thermal cauterotherapy (Agnikarma) in grade V rectal prolapse: a unique case report
Source: Front Med (Lausanne). 2026 Apr 10;13:1761909. doi: 10.3389/fmed.2026.1761909 (PMC13105895; doi:10.3389/fmed.2026.1761909)

**APPENDICES**

a)


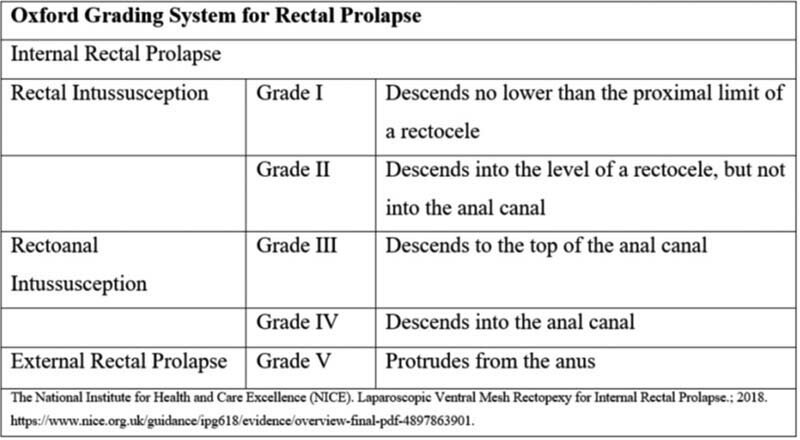


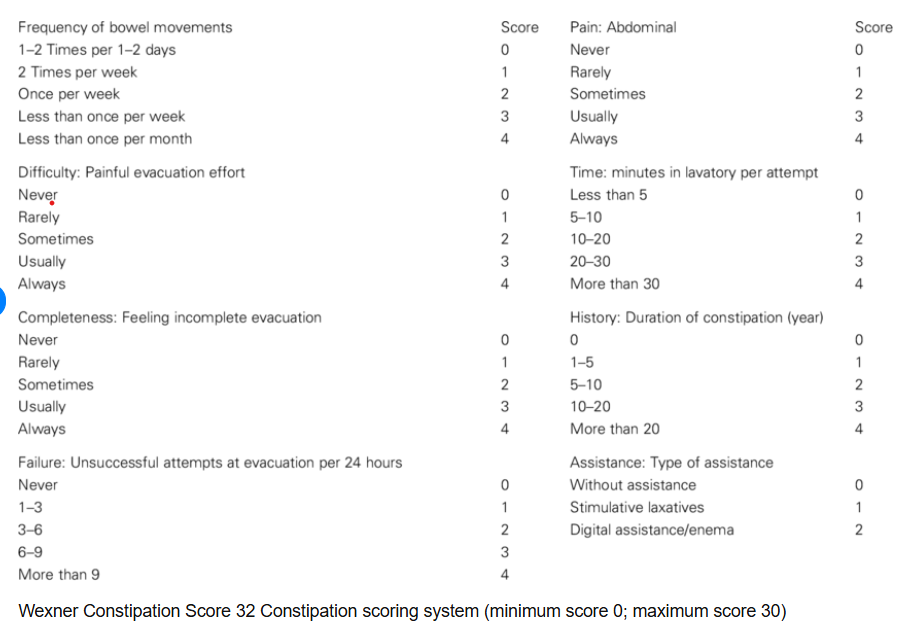
b)


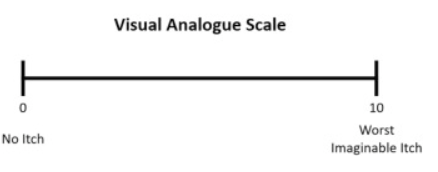
c)

Supplement: Supplementary file 1 [file Table_1.DOCX]
